# Reducing Neonatal Mortality in India: Critical Role of Access to Emergency Obstetric Care

**DOI:** 10.1371/journal.pone.0057244

**Published:** 2013-03-27

**Authors:** Anu Rammohan, Kazi Iqbal, Niyi Awofeso

**Affiliations:** 1 School of Business, Discipline of Economics, University of Western Australia, Perth, Western Australia; 2 School of Population Health, University of Western Australia, Perth, Western Australia; 3 School of Public Health, University of New South Wales, Sydney, Australia; Tehran University of Medical Sciences, Iran (Republic of Islamic)

## Abstract

**Background:**

Neonatal mortality currently accounts for 41% of all global deaths among children below five years. Despite recording a 33% decline in neonatal deaths between 2000 and 2009, about 900,000 neonates died in India in 2009. The decline in neonatal mortality is slower than in the post-neonatal period, and neonatal mortality rates have increased as a proportion of under-five mortality rates. Neonatal mortality rates are higher among rural dwellers of India, who make up at least two-thirds of India's population. Identifying the factors influencing neonatal mortality will significantly improve child survival outcomes in India.

**Methods:**

Our analysis is based on household data from the nationally representative 2008 Indian District Level Household Survey (DLHS-3). We use probit regression techniques to analyse the links between neonatal mortality at the household level and households' access to health facilities. The probability of the child dying in the first month of birth is our dependent variable.

**Results:**

We found that 80% of neonatal deaths occurred within the first week of birth, and that the probability of neonatal mortality is significantly lower when the child's village is closer to the district hospital (DH), suggesting the critical importance of specialist hospital care in the prevention of newborn deaths. Neonatal deaths were lower in regions where emergency obstetric care was available at the District Hospitals. We also found that parental schooling and household wealth status improved neonatal survival outcomes.

**Conclusions:**

Addressing the main causes of neonatal deaths in India – preterm deliveries, asphyxia, and sepsis – requires adequacy of specialised workforce and facilities for delivery and neonatal intensive care and easy access by mothers and neonates. The slow decline in neonatal death rates reflects a limited attention to factors which contribute to neonatal deaths. The suboptimal quality and coverage of Emergency Obstetric Care facilities in India require urgent attention.

## Introduction

Neonatal deaths account for a major proportion of the world's paediatric deaths. Global neonatal mortality declined from 32 deaths per 1,000 live births in 1990 to 23 in 2010 – an average of 1.7 percent a year, much slower than the fall in the under-five mortality rate of 2.2 percent per year. Consequently, the proportion of deaths in the neonatal period rose from 38% (4 million) of total deaths in 2000 to about 41% (3.3 million) in 2009 [1]–[2]. The relatively larger decline in the post-neonatal period compared to the neonatal period may be attributable to the relatively high emphasis and global support for Primary Health Care workforce development and programs such as nutrition, vaccination and health promotion, relative to hospital-related workforce and infrastructure investments that are necessary for neonatal mortality reduction, particularly in rural areas [3]. Neonatal deaths are mainly caused by pre-term birth, asphyxia, sepsis, pneumonia, congenital anomalies and diarrheal diseases.

In this paper we use data from India's 2008 nationally representative *District Level Household Survey* (DLHS-3) to analyse the links between neonatal mortality, household's socio-economic characteristics, and access to health infrastructure and appropriately skilled neonatal healthcare workers.

India presents a unique context to study neonatal mortality for several reasons. First, despite the rapid economic growth that has occurred there over the last two decades, neonatal deaths fell modestly from 1.3 million in 1990 to 875,000 in 2010, and India currently accounts for nearly 28% of the global deaths among newborn children [4]–[5]. Second, figures from India's nationally representative National Family Health Survey (NFHS) datasets show that neonatal deaths have increased as a proportion of under-five deaths from 45% in NFHS-1 (1992) to 52% in NFHS-3 (2005–06), a large increase compared to global figures which show an increase from 37% in 1990 to 41% in 2008. This is despite the fall in under-five mortality rate from 109/1000 live births in NFHS-1 (1992) to 74/1000 live births in the 2005/6 NFHS-3. The focus of the social sciences literature on child survival outcomes in India, has predominantly been on the role of socio-economic factors in influencing infant or child mortality outcomes, with little or no focus on neonatal mortality [6]–[9]. These studies show that variables such as parental education, child's gender, sibling effects, birth-spacing, economic characteristics, religion and caste are important in influencing child mortality outcomes.

However, in India prematurity and low birth weight, infections and birth asphyxia accounted for 78% of all neonatal deaths in 2005 [4]–[5]. Improved survival of neonates from these causes requires the availability and adequacy of specialised maternal and child health-care personnel, and easy access to obstetric and neonatal facilities for pregnant women and newborns. The 2006 World Health Report stresses the positive correlation between infant, child and maternal survival probabilities and a higher density of competent health workers and adequate health infrastructure [10].

Recent research shows that in high mortality settings, access to emergency obstetric care has the greatest effect in improving neonatal survival outcomes, and that lack of access to emergency obstetric care services in low-income countries is a serious constraint in improving pregnancy outcomes [11]–[15]. This is consistent with the situation in India where, although the government of India has implemented programs such as the *Janani Surakhsha Yojana* (JSY) and “Home-based New Born Scheme” to improve maternal and child birth outcomes, their impact on reducing neonatal deaths has been limited [16], [17].

These findings confirm an earlier analysis by Kumar *et* *al* [18] who report the results of a community-based strategy, where the researchers designed and implemented a project called *Saksham* (Empowered) in Uttar Pradesh, India's largest state which accounts for a quarter of all newborn deaths in India. The project was supported by a well-functioning emergency obstetric care system that included dedicated obstetricians, neonatologists, culturally and technically competent community health workers and nurses who organized the referral system from communities to respective district hospitals. Their analysis found that within 18 months of the program's commencement, neonatal deaths dropped by 58%. Therefore, the evidence from the medical literature suggests that while post-neonatal survival outcomes can be influenced by socio-economic factors and access to vaccinations and other public health measures, access to emergency obstetric and neonatal services is critical in order to reduce neonatal deaths.

For India, the links between neonatal-specific care such as access to emergency obstetric care and health infrastructure on neonatal survival probability remains understudied at the national level. A key contribution of our paper is to address this gap in the literature using a large nationally representative dataset that quantifies the role of access health facilities on neonatal mortality outcomes, in a model that also controls for household's socio- economic characteristics.

India's Public Health system has been developed over the years as a 3-tier system, at the primary, secondary and tertiary level of health care. A typical Indian state is divided into a number of districts and the districts in turn are divided into Blocks. The district health system is the fundamental basis for implementing various health policies, delivery of healthcare and the management of health services for a defined geographic area [19]. Every district is expected to have a district hospital linked with the public hospitals/health centres such as Sub-district/Sub-divisional hospitals, Community Health Centres (CHCs), Primary Health Centres (PHC) and Sub-centres (SC). A district hospital is the tertiary referral health centre in India's rural health service. According to the Ministry of Health and Child Welfare [20], the role of the District Hospital (DH) is to provide effective, affordable health care services (curative including specialist services, obstetric and neonatal services and preventive health services) for a defined population.

Table 1 provides a snapshot of the health infrastructure available at the district level in India's rural areas.

**Table 1:** Health infrastructure at the district level.

	Population norm	Human resource available
District hospital	2–3 million	Obstetrician, Anaesthetist, Pathologist, Pediatrician, General doctors, nurses
Community Health Centre (CHC)	100,000–300,000	Any specialist, General doctors, nurses
Primary Health Centre (PHC) at the Block level	100,000	General doctors (2), nurses, LHVs, ANMs

Source: Ministry of Health and Child Welfare, 2010.

In relation to neonatal mortality prevention, skilled workforce entails adequate quality, quantity and distribution of neonatologists, obstetricians, anaesthetists and midwives. Good emergency obstetric care requires improving the availability, accessibility, quality and use of services for the treatment of complications that arise during pregnancy and childbirth [21]. The weakest link in India's Emergency Obstetric Care Services is the provision of well-functioning and appropriately staffed district and referral hospitals to provide care for complications that arise during late pregnancy and at birth. Even in Indian states where such facilities are provided, delays in obtaining care may occur at three levels: delay in deciding to seek care; delay in reaching a first referral level facility, and; delay in actually receiving care after arriving at the facility [22].

As at 2011, there was a 64% shortage of specialist health workers in rural health facilities nationally, including a 66% shortage of Obstetricians and Gynaecologists, and 74% shortage of paediatricians relative to requirements for existing infrastructure [23]–[24]. Critical shortage of neonatal specialists is more severe than that of the general health services, with rural areas facing more shortages than in urban areas. For example, a study of 44 public hospital facilities to determine the adequacy of neonatal and maternal care infrastructure in the relatively rich state of Maharashtra, found that only 45% had a qualified obstetrician/s, 30% had a qualified anaesthetist/s while 77% do not have either or both of these specialists [25]. This article focuses on the impact of distance between a pregnant woman's residence and the nearest District Hospital in relation to neonatal survival.

## Methods

The analysis in this paper is based on the 2008 *District Level Household Survey (DLHS-3),* collected by the International Institute for Population Sciences (IIPS), Mumbai on behalf of the Ministry of Health and Family Welfare (MoHFW), Government of India. The DLHS-3 provides a large sized 5-year retrospective collection of statistical records on maternal and child health practice and outcomes, along with demographic and economic information on both individual mothers and their respective households. There were separate questionnaires for Primary Health Centres (PHC), Community Health Centres (CHC) and District Hospitals (DH), which broadly include questions on infrastructure and human resources.

The survey used two-stage stratified random sampling in rural and three-stage stratified sampling in urban areas of each district. The information from the 2001 Census was used as a sampling frame for selecting primary sampling units (PSUs). For the first time, a population-linked facility survey has been conducted in DLHS-3.

The data on neonatal deaths comes from the ever- married women's questionnaire, with detailed information on the pregnancy history of women, if any child was born alive but died subsequently, information on the age and sex of the child at the time of death, and information on maternal access to prenatal and postnatal care, and details regarding the place of delivery. This questionnaire also contains detailed information on the socio-economic, demographic and labour market characteristics of the respondent's household. Similarly the village questionnaire collects data from the village head on village infrastructure and the access of the village to health facilities. The DH, the PHC and the CHC questionnaires similarly contain detailed information on infrastructure and health workforce availability and the skills available at each of these health institutions. We linked this detailed mother-level data to information on availability, access and services to health infrastructure, using the Village questionnaire, the DH questionnaire, the PHC questionnaire and the CHC questionnaire.

Our analysis is based on 99,735 rural women who gave birth in the last 5 years, and for whom data is available on all our variables of interest. We focus on the last pregnancy since information on birth related characteristics is only available for the last birth. –Similarly we restrict our analysis to the rural sample since data on village-specific characteristics is only available for the rural sample, as 75% of the Indian population live in rural areas, neonatal mortality rates are higher in rural than in urban areas of India and finally, access to health infrastructure is likely to be a bigger constraint in rural areas.

In Table 2 we present the descriptive statistics for the key variables used in the analysis, disaggregated for the full sample and for the sample of children who died in the neonatal period. The Table presents the sample mean of the proportion of children who fall in According to the descriptive statistics presented in Table 2, approximately 2% of the children died in the neonatal period, representing 20 neonatal deaths per 1000 live births. Since our data on neonatal deaths is from the mother's questionnaire, we do not have any information on children whose mothers died at childbirth. Given that maternal mortality rates are high in India, it is likely that the neonatal deaths in our sample may well be an underestimation.

**Table 2:** Selected descriptive statistics.

Variable	Full sample (99,735)	Neonatal death (2003)
Neonatal = 1 if child is born alive but died within a month of birth	0.02	1
Distance from Village to DH (kms)	38.72	39.13
Delivery room at DH – dummy variable = 1 if yes, 0 otherwise	0.84	0.82
Emergency obstetric care at DH = 1 if gynaecologist is available 24 hrs at DH	0.83	0.80
Average number of Paediatrician in a district	1.98	2.01
Distance from Village to CHC (km)	18.27	17.94
Average number of gynaecologist at CHCs in a Block	0.26	0.28
Average number of paediatrician at CHCs in a Block	0.18	0.21
Operation theatre at CHC – dummy variable = 1 if yes,	0.57	0.63
Operation theatre PHC – dummy variable = 1 if yes,	0.45	0.46
Num. of referrals as a share of delivery performed in last year	0.27	0.23
Distance from Village to PHC (kms)	9.64	9.25
Respondent's religion: Hindu- dummy variable = 1 if yes	0.81	0.82
Respondent's religion: Muslim- dummy variable = 1 if yes	0.13	0.13
Respondent's caste: Scheduled caste/tribe – dummy variable = 1 if yes	0.36	0.35
Wealth quintile- poorest – reference group	27	31
Wealth quintile- poor – dummy variable = 1 if yes	0.26	0.29
Wealth quintile- middle- dummy variable = 1 if yes	0.22	0.21
Wealth quintile- rich- dummy variable = 1 if yes	0.17	0.14
Wealth quintile- richest- dummy variable = 1 if yes	0.08	0.05
Father ever attended school- dummy variable = 1 if yes	0.71	0.66
Mother ever attended school	0.45	0.39
Mother's age at birth	24.97	24.51
Multiple birth – dummy variable = 1 if yes	0.009	0.05
Male	0.53	0.58
Birth order second	0.25	0.19
Birth order third	0.17	0.13
Birth order fourth	0.10	0.09
Birth order fifth and above	0.17	0.19
Problems during birth: premature labour- dummy variable = 1 if yes	0.31	0.34
Problems during birth: excessive bleeding- dummy variable = 1 if yes	0.08	0.11
Problems during birth: prolonged labour- dummy variable = 1 if yes	0.21	0.25
Problems during birth: obstructed labour- dummy variable = 1 if yes	0.45	0.48
Problems during birth: breech presentation- dummy variable = 1 if yes	0.04	0.07
Problems during birth: convulsion/high b.p- dummy variable = 1 if yes	0.04	0.05

Note: The table reports the mean proportion falling in each category for indicator variables and the mean for continuous variables.

Comparing the full sample with the sample of children who died in the neonatal period, we observe that 84% of the full sample had access to a delivery room in the district hospital, but the figure is slightly lower at 82% in the neonatal death sample. Similarly, a higher proportion of the households (83%) had access to emergency obstetric care in the full sample, compared to the sample of neonatal deaths (80%).

We control for household characteristics such as the household head's religion, the mother's age at birth, and the birth-order of the child, such as whether the child was the first, second or third and higher parity birth. The dataset does not have any information on the household's income, wages or expenditure. Hence, we use the wealth index that is available in the dataset. We used the wealth index which was available in the dataset. The wealth index was constructed using household asset data, and is divided into five population quintiles. Notably children from the poorest wealth quintile are over-represented in the sample of neonatal deaths, compared to the full sample.

Turning to the role of parental education, in general we observe that education levels are low for mothers. The schooling for fathers albeit higher, shows some disparity between the full sample and the sample of neonatal deaths. While only 45% of the mothers have ever attended school in the full sample, the figure is 71% for the fathers in our study. Among children who died in the neonatal period, only 39% of the mothers have ever attended school. It is also noteworthy that relative to the full sample, a higher proportion of children who died in the neonatal period were also more likely to have had health problems during childbirth.

We estimate the probability of a child dying in the neonatal period using a reduced form binary choice probit model as below:
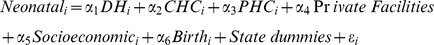
where the term 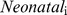, our dependent variable is an indicator variable which takes on a value of 1 if a child is born alive but died within a month of birth, 0 otherwise.

Our key explanatory variables include socio-economic variables and an array of health infrastructure variables guided by the medical literature on services critical to neonatal care in India. The term 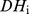 is a vector of variables on the characteristics of the District Hospitals (DH), and includes variables such as the distance to the DH from household *i*'s village, whether there is a delivery room at the DH, the availability of trained health personnel at the DH, in particular the availability of emergency obstetric care (24-hour gynaecologist/obstetrician), and the availability of a paediatrician at the DH. We assume that household *i* can only access health facilities in their district.

The vectors CHC and PHC include variables at the lower referral hospital. As with the DH, to capture information on the availability of health personnel, we include two variables- the average number of gynaecologists and the average number of paediatricians available at Block-level CHCs. We also include dummy variables indicating the availability of operation theatres at the CHC and at the PHC. Since the PHC acts as the first point of referral, we create a variable PHC_ref_del indicating the average number of referrals to the DH as a ratio of the number of deliveries performed at the PHCs within a Block. There is no information in the household dataset on the specific CHC or PHC that the household may visit, we assume therefore that the household has access to all the CHCs and PHCs in their Block, and accordingly include the average number of the health personnel and infrastructure of all CHCs and PHCs in the Block. A full description of all the health infrastructure variables included in the analysis is provided as Appendix S1.

Households may also potentially have access to private health facilities such as private clinics and hospitals. Given that the DLHS does contain information on health infrastructure and personnel in private health facilities, we include a variable indicating the average distance between the nearest private health facilities to the respondent's village.

The term *Socioeconomic* refers to household characteristics such as the wealth quintile, a dummy variable for whether or not the child's mother and father have attended school, and variables to indicate the household head's religion and caste.

The term *Birth* includes the child's birth-specific characteristics such as their gender, their birth-order, whether the child is part of twin births, dummy variables for specific birth-related complications such as whether it was a breech delivery, whether the labour was prolonged, etc., and mother-specific variables such as her age at birth. We also include dummy variables for states to control for time in-variant state-specific unobserved heterogeneity, and finally 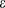 refers to the error term.

The source of identification in our sample is the cross-sectional variation across Blocks and districts. The data on PHCs and CHCs are available at the Block level, and DH data are available at the district level, and thus vary only across districts, although we note that some of the districts have more than one district hospital, in which case we have assumed that the mother would go to the nearest DH.

The health facilities that we have included among our explanatory variables are fairly exogenous to the health-seeking behaviour of households. All the household specific socio-economic and birth-related characteristics are also exogenous to household decisions. However, one can argue that some unobserved health attributes may lead to the household being in a higher wealth quintile and also the probability of having a healthy baby. We have included the household's wealth rather than income given that it is less likely to be endogenous.

Furthermore, there are variables in the questionnaire on whether the mother received antenatal and post-natal care, if skilled health personnel were present at the time of delivery, whether the child's birth was at an institutional facility, whether the mother received any assistance from government health-related programs (such as JSY). These variables are likely to have a direct bearing on neonatal death. However, these decision variables are the outcome of an interplay between supply-side variables (such as access to health infrastructure and personnel), child-specific birth characteristics, and also the household's socioeconomic characteristics which we have already included in the regression model. In the presence of both supply and demand-side variables, variables relating to care, skilled personnel and program participation become redundant as the former influences the latter. Similarly, regional unobservable characteristics have the potential to confound the impact of supply side variables, and as discussed the inclusion of state level dummy variables control for state-specific unobserved heterogeneity.

## Results and Discussion

Our estimation results are presented in Tables 3 and 4. Table 3 reports the probit marginal effects and robust standard errors in parentheses for the full sample results of our analysis can be summarised as follows: (i) the probability of neonatal mortality was significantly lower when the child's village is closer to the district hospital (DH); (ii) neonatal deaths were lower in regions where emergency obstetric care is available at the District Hospitals; (iii) the availability of services at lower level referral hospitals such as the Community Health Centres (CHC) and the Primary Health Centres (PHCs) were statistically insignificant in influencing neonatal mortality outcomes; and (iv) variables relating to parental schooling and household wealth status were found to improve neonatal survival outcomes.

**Table 3:** Probit estimation results: Dependent variable- Probability of child dying in the neonatal period.

VARIABLES	ME	Std. Errors	ME	Std.Errors
	Full sample		Backward states	
Distance of village from DH	0.0001*	(0.0000)	0.0001**	(0.0000)
Delivery room at DH	−0.0009	(0.0013)	−0.0008	(0.0016)
24-hr availability of gyn at DH	−0.0019	(0.0013)	−0.0016	(0.0015)
Paediatrician at DH	−0.0004	(0.0003)	−0.0007*	(0.0004)
Distance of village from CHC	−0.0000	(0.0000)	−0.0000	(0.0000)
Gynaecologists at CHC	−0.0002	(0.0009)	0.0001	(0.0011)
Paediatrician at CHC	0.0019*	(0.0011)	0.0019	(0.0013)
Operation theatre at CHC	0.0010	(0.0011)	0.0002	(0.0014)
Referral as a prop of delivery at PHC	−0.0000	(0.0001)	0.0002	(0.0004)
Operation theatre at PHC	0.0001	(0.0009)	−0.0004	(0.0011)
Distance of village from PHC	−0.0001**	(0.0001)	-0.0002**	(0.0001)
Distance to private clinic/hospital	−0.0000	(0.0000)	−0.0000	(0.0000)
Hindu	0.0027	(0.0024)	0.0014	(0.0041)
Muslim	0.0033	(0.0033)	0.0014	(0.0046)
Scheduled caste/tribe	0.0005	(0.0009)	−0.0009	(0.0012)
Wealth quintile- poor	−0.0002	(0.0011)	−0.0006	(0.0013)
Wealth quintile- middle	−0.0021*	(0.0012)	−0.0030**	(0.0014)
Wealth quintile- rich	−0.0040***	(0.0013)	-0.0043***	(0.0016)
Wealth quintile- richest	−0.0074***	(0.0014)	-0.0071***	(0.0020)
Father ever attended school	−0.0015	(0.0010)	−0.0018	(0.0012)
Mother ever attended school	−0.0013	(0.0010)	−0.0012	(0.0012)
Mother's age at birth	−0.0018***	(0.0006)	-0.0019***	(0.0007)
Mother's age at birth-square	0.0000***	(0.0000)	0.0000***	(0.0000)
Multiple birth	0.0842***	(0.0101)	0.1015***	(0.0126)
Male	0.0038***	(0.0008)	0.0048***	(0.0010)
Birth order second	−0.0079***	(0.0009)	-0.0091***	(0.0011)
Birth order third	−0.0086***	(0.0010)	-0.0101***	(0.0012)
Birth order fourth	−0.0071***	(0.0012)	-0.0088***	(0.0014)
Birth order fifth and above	−0.0061***	(0.0013)	-0.0083***	(0.0016)
Problems: premature labour	0.0000	(0.0009)	−0.0007	(0.0011)
Problems: excessive bleeding	0.0057***	(0.0017)	0.0078***	(0.0021)
Problems: prolonged labour	0.0017	(0.0011)	0.0021	(0.0013)
Problems: obstructed labour	−0.0003	(0.0009)	−0.0004	(0.0011)
Problems: breech presentation	0.0114***	(0.0025)	0.0134***	(0.0031)
Problems: convulsion/high b.p	0.0011	(0.0020)	0.0001	(0.0023)
State dummy variables	Yes		Yes	
Observations	99,735		76,072	

Standard errors are in parentheses. *** p<0.01, ** p<0.05, * p<0.1.

**Table 4:** Robustness checks.

	Robustness check 1	Robustness check 2	Robustness check 3
	Prob that child died in week 1: full sample	Prob that child died in neonatal period: sample of mothers who had institutional delivery	Prob that child died in neonatal period: sample of mothers who had accessed prenatal care
Distance of village from DH	0.0001***(0.0000)	0.00004*(0.0000)	0.00004** (0.000)
Delivery room at DH	−0.0010 (0.0012)	−0.0009 (0.0016)	−0.0018 (0.0013)
24-hr availability of gyn at DH	−0.0011(0.0012)	−0.0017(0.0020)	−0.0020*(0.0012)
Paediatrician at DH	−0.0003(0.0002)	−0.0004(0.0004)	−0.0003(0.0003)
Distance of village from CHC	−0.0000(0.0000)	0.0001(0.0001)	−0.0000
Gynaecologists at CHC	−0.0000(0.0008)	0.0006(0.0010)	0.0006 (0.0009)
Paediatrician at CHC	0.0009(0.0010)	0.0020 (0.0019)	0.0020*(0.0012)
Operation theatre at CHC	0.0011(0.0010)	0.0016(0.0016)	−0.0001(0.0015)
Referral as a share of delivery (PHC)	−0.0000(0.0001)	−0.0001(0.0001)	−0.0000(0.0001)
Operation theatre at PHC	−0.0002(0.0008)	0.0014 (0.0015)	0.0006 (0.0012)
Distance of village from PHC	−0.0001*(0.0001)	−0.0001(0.0001)	–0.0001**(0.0001)
Distance to pvt clinic/hospital	−0.0000(0.0000)	−0.0000(0.0000)	−0.0000(0.0000)
State dummy variables	Yes	Yes	Yes
Observations	99,735	34,781	68,483

Standard errors are in parentheses. *** p<0.01, ** p<0.05, * p<0.1. As in Table 3, all socioeconomic and birth related characteristics are included.

Table 3 presents Probit estimation results for the full sample (columns 1 and 2) and for the sample of backward states in columns 3 and 4 (states with GDP per capita below the national average) respectively. A backward state is defined as one with a GDP per capita below the national average of $1450. These are the states of West Bengal, Assam, Bihar, Chhattisgarh, Jharkhand, Madhya Pradesh, Rajasthan, Orissa, Uttar Pradesh, and Jammu and Kashmir.

The dependent variable neonatal mortality is an indicator variable (0,1) for the probability of a child being born alive, but dying in the first month. We report marginal effects and robust standard errors.

Our analysis indicates a statistically significant and negative association between variables relating to district hospitals and neonatal survival outcomes. For both full sample and the backward states sample, the variable distance to DH was statistically significant and positive. Specifically, according to Table 3 (Columns 2–4) if a household lives one kilometre closer to the district hospital, the probability of neonatal death decreases by 0.01 percent. That is, if the services of DHs are brought 10 km closer to the village, it can save one more child out of 1000 births. Note that the average distance of a village from a DH is about 39 km for the full sample. Poor rural transport facilities for pregnant women constitute a major encumbrance to accessing antenatal services and emergency obstetric care in India and internationally [26]–[27].

However, distance to PHC is found to be negative and statistically significant for the full sample result. In other words, an increase in the distance between the respondent's household and PHC reduces the probability of neonatal death. Although this result may appear counter intuitive, the negative association may simply imply that if the PHC is closer, the mother may choose to deliver at home or take the child to the PHC which is ill-equipped to deal with neonatal care, and the probability of a child dying in the neonatal period increases. This result is consistent with evidence that these PHCs are not well equipped to deal with complications in neonatal care.

The availability of emergency obstetric care (measured using the variable 24-hour availability of obstetrician/gynaecologist) is not statistically significant when state level dummy variables are included. This maybe because it is unclear from our dataset whether it is the obstetrician or the gynaecologist who is available for 24 hours, and they perform different roles. Moreover, the state dummy variables may capture state-specific observed and unobserved characteristics that affect neonatal death such as the quality of health care service. While other measures of health facilities only capture the number of health professionals in the facilities, the emergency obstetric care variable captures whether these health professionals are available for 24 hours. The above results highlight the fact that access to services at the DH, particularly access to emergency obstetric care is crucial in reducing neonatal deaths, particularly in India's less developed states. Although the lower level hospitals such as the CHC and PHC might have a role in referring cases to the DH, they do not appear to have any direct influence.

With regards to child characteristics, the results broadly accord with those found in the literature. In particular, relative to a female child, a male child has a significantly higher probability of dying in the first month. The child's birth order is also statistically significant. Relative to a first-born child, higher birth order children have a significantly lower probability of dying in the neonatal period. This may be because more experienced mothers may be in a better position to pick the danger signs during pregnancy. These results hold both for the full sample and in the sample of backward states. Similarly, a child who is part of a twin has a significantly lower probability of survival compared to a singleton birth. We also include variables relating to whether the birth was complicated, and our results show that the probability of neonatal death is significantly increased when there was excessive bleeding and breech presentation at the time of the child's birth. Both conditions are considered to be common causes of birth asphyxia, a known risk factor for neonatal death.

Not surprisingly, having a mother who has some schooling relative to none significantly lowers the probability of neonatal death. Our results also show that relative to a child from the lowest wealth quintile, children from the highest three wealth quintiles have a significantly lower probability of dying in the neonatal period. Mother's age has a non-linear relationship with neonatal mortality, indicating that the probability of neonatal morality increases with mother's age. The variables religion and schedule caste/tribe are not statistically significant. This may be because the wealth quintile captures much of these differences and also the Government of India has already introduced a number of programs such as JSY that improve access to health services for disadvantaged people.

Finally, we have included controls for states to account for state-specific differentials in neonatal mortality. The full set of results are not presented in Table 3 due to space considerations, but are included Table S1. These results indicate that relative to children from the state of Tamil Nadu (reference category), children in the states of Jammu and Kashmir, Rajasthan, Uttar Pradesh, Bihar, Assam and Madhya Pradesh, have a significantly higher probability of dying in the neonatal period. It is noteworthy that these states are among the poorest in the country and our results show that despite controlling for household wealth, the probability of neonatal mortality is higher for children from these relatively poorer states where the quality of health personnel may be poor or if access to health infrastructure is lower. On the other hand, the probability of neonatal deaths is lower for children from the states of Punjab, Haryana and Maharashtra, relative to children from the state of Tamil Nadu.

**Figure 1:**
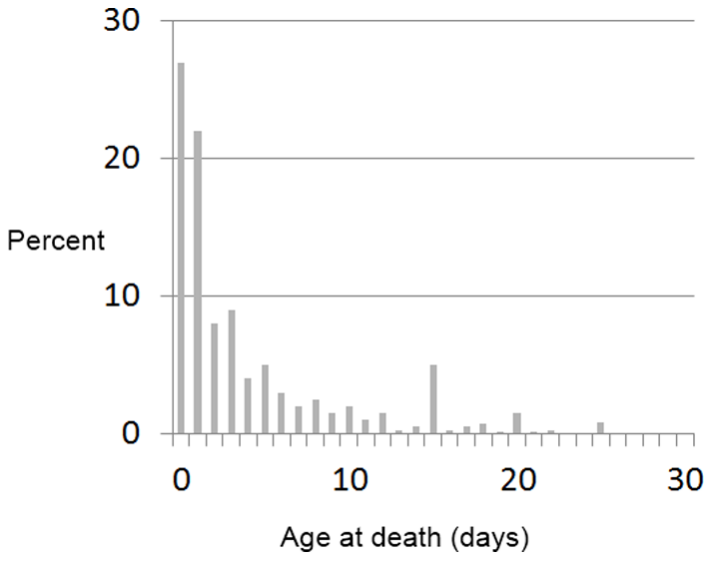
Breakdown of neonatal sample by age at death (in days) following birth.

We test the robustness of our results to alternative specifications. First, previous research has suggested that the likelihood of neonatal mortality is highest in the first week of birth [27]–[28]. In our sample, 80 percent of neonatal deaths occurred in the first week of birth (Figure 1).

Therefore, as a first robustness check we re-estimate a model where the dependent variable is defined as the probability of a child dying in the first week of birth, using the same set of explanatory variables as in the previous regressions (Table 4, Robustness check 1). The second robustness check that we conduct is to test if these results hold for the sample of children who were born at an institutional facility (Robustness check 2 in Table 4) and the third robustness check results that we present only considers the sample of children whose mothers accessed prenatal care (Robustness check 3 in Table 4). We have also estimated a model where we have included interaction.

Our results show that the distance to the DH is statistically significant and positively signed for this sample, albeit the size of the marginal effects are small. This result holds for all the three robustness tests.

As a final robustness check, we considered if there was a link between the nearest health facility, health personnel and infrastructure. To assess this, we include two interaction dummy variables between distance and health facilities (e.g., operation theatre) and distance and health personnel (e.g., gynaecologists). The interaction terms are not significant in any of our specifications and we have chosen not to report them due to space considerations. These results are however presented in Appendix S1.

## Conclusions

India accounts for the largest number of global neonatal deaths with 900,000 of 3.3 million neonatal deaths in 2010. Despite high economic growth rates over the last two decades and declines in child mortality rates, neonatal mortality rates remain high and have increased as a proportion of child mortality rates. Since 1995, the Indian government has instituted two major public health programs to improve maternal and child survival outcomes (i.e. *Janani Surakhsha Yojana* and “Home-based New Born Scheme”), but these initiatives have not succeeded in significantly reducing deaths in the neonatal period. The medical literature has highlighted the importance of emergency obstetric care in reducing neonatal mortality. In this paper, using nationally representative data we examined the links between neonatal mortality and access to health infrastructure, particularly focusing on the role of distance various health care facilities. Our results show that the probability of neonatal death is lower if the household lives closer to the DH, which is the only health facility with emergency obstetric care. Access to lower level health facilities such as CHCs and PHCs, are shown to be insignificant in influencing neonatal mortality outcomes.

The above results have significant implications for policies aiming at reducing neonatal deaths in India. We demonstrate that despite the growing interest in community-based delivery platforms for maternal and newborn care, many of these interventions will remain ineffective in reducing neonatal mortality without well-equipped emergency obstetric care in district hospitals and availability of an appropriate mix of neonatologists, obstetricians and midwives in India's rural hospitals.

## Supporting Information

Appendix S1 **Description of supply side variables.**
(DOCX)

Table S1 **Probit estimation results: Dependent variable- Probability of child dying in the neonatal period.**
(DOCX)
