# Supplementary material for: Reducing Neonatal Mortality in India: Critical Role of Access to Emergency Obstetric Care
Source: PLoS One. 2013 Mar 27;8(3):e57244. doi: 10.1371/journal.pone.0057244 (PMC3609864; doi:10.1371/journal.pone.0057244)
Supplement: Appendix S1 — Description of supply side variables. (DOCX) [file pone.0057244.s001.docx]

**Appendix S1: Description of supply side variables**

District Hospital delivery room: A measure of the average number of district hospitals in a district with a functioning delivery room. Variable taken from the District Hospital survey, 1 indicates that a delivery room is available, and 0 otherwise. If a household has access to 2 district hospitals, one with a delivery room and one without, then the value of this variable will be 0.5.

District Hospital gynaecologists available 24 hours: The number of district hospitals in a district with 24 hours gynaecologist availability. Mean of the dummy variables- 1 if yes and 0 otherwise.

District Hospital paediatrician: Average number of paediatricians available in district hospitals, both in position and on contract.

CHC gynaecologists: Average number of gynaecologists available in CHC in a Block.

CHC paediatrician: Average number of paediatrician available in in CHC in a Block.

CHC operation theatre: A measure of the average number of CHCs in a Block with functional operation theatres. If a person has access to 2 CHCs where one has functional operation theatres while the other doesn’t then the value of this variable will be 0.5.

PHC operation theatre: It is a measure of the average number of PHCs with functional operation theatres that in a Block. This is the mean of dummy variables which takes on values 1 and 0. If a person has access to 3 CHCs where one has functional operation theatres while the other two don’t then the value of this variable will be 0.33.

PHC referral delivery: It is the number of delivery cases referred to higher facilities (CHC, DH) as a ratio of delivery performed at the PHC.
