# Supplementary material for: Reducing Neonatal Mortality in India: Critical Role of Access to Emergency Obstetric Care
Source: PLoS One. 2013 Mar 27;8(3):e57244. doi: 10.1371/journal.pone.0057244 (PMC3609864; doi:10.1371/journal.pone.0057244)
Supplement: Table S1 — Probit estimation results: Dependent variable- Probability of child dying in the neonatal period. (DOCX) [file pone.0057244.s002.docx]

**Supplementary Appendix**

**Table S1: Probit estimation results: Dependent variable- Probability of child dying in the neonatal period**

| **VARIABLES** | **ME** | **Std. Errors** | **ME** | **Std.Errors** |
| --- | --- | --- | --- | --- |
|  | **Full sample** | | **Backward states** | |
| Distance of village from DH | 0.0001* | (0.0000) | 0.0001** | (0.0000) |
| Delivery room at DH | -0.0009 | (0.0013) | -0.0008 | (0.0016) |
| 24-hr availability of gyn at DH | -0.0019 | (0.0013) | -0.0016 | (0.0015) |
| Paediatrician at DH | -0.0004 | (0.0003) | -0.0007* | (0.0004) |
| Distance of village from CHC | -0.0000 | (0.0000) | -0.0000 | (0.0000) |
| Gynaecologists at CHC | -0.0002 | (0.0009) | 0.0001 | (0.0011) |
| Paediatrician at CHC | 0.0019* | (0.0011) | 0.0019 | (0.0013) |
| Operation theatre at CHC | 0.0010 | (0.0011) | 0.0002 | (0.0014) |
| Referral as a prop of delivery at PHC | -0.0000 | (0.0001) | 0.0002 | (0.0004) |
| Operation theatre at PHC | 0.0001 | (0.0009) | -0.0004 | (0.0011) |
| Distance of village from PHC | -0.0001** | (0.0001) | -0.0002** | (0.0001) |
| Distance to private clinic/hospital | -0.0000 | (0.0000) | -0.0000 | (0.0000) |
| Hindu | 0.0027 | (0.0024) | 0.0014 | (0.0041) |
| Muslim | 0.0033 | (0.0033) | 0.0014 | (0.0046) |
| Scheduled caste/tribe | 0.0005 | (0.0009) | 0.0009 | (0.0012) |
| Wealth quintile- poor | -0.0002 | (0.0011) | -0.0006 | (0.0013) |
| Wealth quintile- middle | -0.0021* | (0.0012) | -0.0030** | (0.0014) |
| Wealth quintile- rich | -0.0040*** | (0.0013) | -0.0043*** | (0.0016) |
| Wealth quintile- richest | -0.0074*** | (0.0014) | -0.0071*** | (0.0020) |
| Father ever attended school | -0.0015 | (0.0010) | -0.0018 | (0.0012) |
| Mother ever attended school | -0.0013 | (0.0010) | -0.0012 | (0.0012) |
| Mother’s age at birth | -0.0018*** | (0.0006) | -0.0019*** | (0.0007) |
| Mother’s age at birth-square | 0.0000*** | (0.0000) | 0.0000*** | (0.0000) |
| Multiple birth | 0.0842*** | (0.0101) | 0.1015*** | (0.0126) |
| Male | 0.0038*** | (0.0008) | 0.0048*** | (0.0010) |
| Birth order second | -0.0079*** | (0.0009) | -0.0091*** | (0.0011) |
| Birth order third | -0.0086*** | (0.0010) | -0.0101*** | (0.0012) |
| Birth order fourth | -0.0071*** | (0.0012) | -0.0088*** | (0.0014) |
| Birth order fifth and above | -0.0061*** | (0.0013) | -0.0083*** | (0.0016) |
| Problems: premature labour | 0.0000 | (0.0009) | -0.0007 | (0.0011) |
| Problems: excessive bleeding | 0.0057*** | (0.0017) | 0.0078*** | (0.0021) |
| Problems: prolonged labour | 0.0017 | (0.0011) | 0.0021 | (0.0013) |
| Problems: obstructed labour | -0.0003 | (0.0009) | -0.0004 | (0.0011) |
| Problems: breech presentation | 0.0114*** | (0.0025) | 0.0134*** | (0.0031) |
| Problems: convulsion/high b.p | 0.0011 | (0.0020) | 0.0001 | (0.0023) |
| Rajasthan | 0.0139** | (0.0064) | 0.0056** | (0.0027) |
| Uttar Pradesh | 0.0108** | (0.0050) | 0.0129*** | (0.0024 |
| Bihar | 0.0175*** | (0.0048) | 0.0037 | (0.0023) |
| Assam | 0.0090** | (0.0045) | 0.0002 | (0.0026) |
| West Bengal | 0.0056 | (0.0044) | -0.0007 | (0.0031) |
| Jharkhand | 0.0044 | (0.0046) | -0.0050 | (0.0033) |
| Orissa | 0.0003 | (0.0046) | -0.0034 | (0.0030) |
| Chattisgarh | 0.0019 | (0.0045) | 0.0072** | (0.0033) |
| Madhya Pradesh | 0.0132** | (0.0057) |  |  |
| Gujarat | 0.0051 | (0.0041) |  |  |
| Maharashtra | -0.0063** | (0.0030) |  |  |
| Andhra Pradesh | -0.0034 | (0.0035) |  |  |
| Karnataka | 0.0095 | (0.0062) |  |  |
| Kerala | 0.0075 | (0.0053) |  |  |
| Jammu and Kashmir | 0.0174** | (0.0066) |  |  |
| Himachal Pradesh | -0.0053 | (0.0034) |  |  |
| Punjab | -0.0124*** | (0.0026) |  |  |
| Uttarakhand | 0.0198** | (0.0081) |  |  |
| Haryana | -0.0100*** | (0.0028) |  |  |
| Observations | 99,735 |  | 76,072 |  |

**Table 1B: Robustness Check 4: Dependent variable: probability of neonatal death**

|  | ME | SE |
| --- | --- | --- |
| Distance of village from DH | 0.0001 | (0.0001) |
| Delivery room at DH | -0.0010 | (0.0017) |
| 24-hr availability of gyn at DH | 0.0013 | (0.0029) |
| Paediatrician at DH | 0.0000 | (0.0006) |
| Distance of village from DH*24-hr availability of gyn at DH | -0.0000 | (0.0001) |
| Distance of village from DH* Paediatrician at DH | -0.0000 | (0.0000) |
| Distance of village from CHC | -0.0000 | (0.0000) |
| Gynaecologists at CHC | -0.0017 | (0.0015) |
| Paediatrician at CHC | 0.0024 | (0.0019) |
| Operation theatre at CHC | 0.0018 | (0.0014) |
| Distance of village from CHC* Gynaecologists at CHC | 0.0001 | (0.0001) |
| Distance of village from CHC* Paediatrician at CHC | -0.0000 | (0.0001) |
| Referral as a share of delivery (PHC) | 0.0000 | (0.0001) |
| Operation theatre at PHC | -0.0009 | (0.0010) |
| Distance of village from PHC | -0.0001* | (0.0001) |
| Distance to pvt clinic/hospital | -0.0001 | (0.0000) |
| State dummy variables | Yes |  |
| Observations | 99735 |  |

Standard errors are in parentheses. *** p<0.01, ** p<0.05, * p<0.1. As in Table 3, all socioeconomic and birth related characteristics are included.
